# Temporal-spatial analysis of hospitalizations for bronchiolitis in Brazil: prediction of epidemic regions and periods for immunization against the Respiratory Syncytial Virus

**DOI:** 10.1590/1984-0462/2023/41/2021304

**Published:** 2023-03-13

**Authors:** Edilenia Queiroz Pereira, Márcia Lorena Alves dos Santos, Taqueco Teruya Uchimura, Eniuce Menezes

**Affiliations:** a Universidade Estadual de Maringá, Maringá, PR, Brazil.

**Keywords:** Bronchiolitis viral, Respiratory syncytial virus infections, Palivizumab, Time series, Bronquiolite viral, Infecções por vírus sincicial respiratório, Palivizumab, Séries temporais

## Abstract

**Objective:**

Due to the high cost and short term of passive immunization against the respiratory syncytial virus, the main virus causing acute viral bronchiolitis, predicting epidemic regions and epidemic months is extremely important. The objective of this study is to identify both the month when the seasonal peak begins and Brazilian regions and states with the highest incidence of monthly hospitalizations due acute viral bronchiolitis.

**Methods:**

Based on data obtained from DATASUS, monthly hospitalization rates due acute viral bronchiolitis were calculated for every 10,000 live births to children under 12 months of age in all Brazilian states and the Federal District between 2000 and 2019. Seasonal autoregressive integrated moving average models were estimated to forecast monthly hospitalization rates in 2020.

**Results:**

A higher incidence of hospitalizations was found for male children, especially under six months of age. As for Brazilian regions, between 2000 and 2019, the South region registered the highest incidence of hospitalizations, followed by the Southeast, Midwest, North and Northeast regions, in this order. Considering the seasonal peak, the period between March and July 2020 comprised the highest expected hospitalization rates.

**Conclusions:**

Palivizumab is suggested to be started between February/March and June/July for most Brazilian states, with the exception of Rio Grande do Sul, which, in addition to presenting the highest rates of hospitalizations for acute viral bronchiolitis per 10,000 live births, has the longest seasonal peak between May and September.

## RESUMO

**Objetivo:**

Em razão do alto custo e do curto prazo da imunização passiva contra o vírus sincicial respiratório, principal vírus causador de bronquiolite viral aguda, a previsão das regiões e meses epidêmicos é extremamente importante. Objetiva-se identificar o mês de início do pico sazonal e as regiões e Estados brasileiros de maior incidência de hospitalizações mensais por bronquiolite viral aguda.

**Métodos:**

Com dados obtidos no Departamento de Informática do Sistema Único de Saúde do Brasil, foram calculadas as taxas mensais de hospitalizações por bronquiolite viral aguda a cada 10 mil nascidos vivos de crianças com idade inferior a 12 meses, em todos os Estados brasileiros e no Distrito Federal, no período entre 2000 e 2019. Modelos sazonais autorregressivos integrados e de médias móveis foram estimados para a previsão das taxas mensais de hospitalizações em 2020.

**Resultados:**

Verificou-se maior incidência de hospitalizações em crianças do sexo masculino, principalmente naquelas com idade inferior a seis meses. Em relação às regiões brasileiras, entre 2000 e 2019, a Região Sul apresentou a maior incidência de hospitalizações, seguida pelas Regiões Sudeste, Centro-Oeste, Norte e Nordeste, respectivamente. Quanto ao pico sazonal, o período entre março e julho de 2020 compreende as maiores taxas de hospitalizações previstas.

**Conclusões:**

Sugere-se o início da administração do Palivizumab entre fevereiro/março e junho/julho para a maioria dos Estados brasileiros, com exceção do Rio Grande do Sul, que, além de apresentar as maiores taxas de hospitalizações por bronquiolite viral aguda a cada 10.000 nascidos vivos, possui o pico sazonal de maior duração entre maio e setembro.

## INTRODUCTION

Acute viral bronchiolitis (AVB) is the result of inflammation and obstruction of the bronchioles, caused by respiratory viruses. Among viruses that cause AVB, the most common are: respiratory syncytial virus (RSV), adenovirus (ADV), influenza virus types A and B and parainfluenza virus (PIV) types 1, 2 and 3.^1,2^ AVB can also be caused by Mycoplasma pneumoniae, rhinovirus, Chlamydia pneumoniae, human metapneumovirus and coronavirus viruses.^3^ In Brazil, RSV is considered the main cause of mortality of children under 12 months of age^4^, being responsible for 60 to 75% of all cases of AVB.^5^

AVB has a low mortality rate (<1%), although it can be higher (30%) in high-risk groups of children such as premature infants younger than 32 weeks and/or with bronchopulmonary dysplasia, children under two years with congenital heart disease and/or immunocompromised, malnourished,^3,6^ considering that these groups present with prolonged illness and greater risk of death. Other risk factors include early weaning, exposure to cigarette smoke, age below six months, school-aged sibling living in the same household, socioeconomic status, being black and male.^7^

Incidence of viral infections may vary depending on local climatic conditions. AVB, in particular, is a seasonal disease that coincides with epidemics of secondary infections and viral respiratory pathogens.^8^ In this sense, periods with low temperatures contribute to the higher incidence of AVB cases related to RSV infections.^2,9^ Studies report the occurrence of a seasonal peak of RSV infection in the city of Rio de Janeiro from March to May,^10^ in the city of São Paulo from April/May to July/August^11,12^ and, in Salvador, between May and July.^13^ The seasonal peak of RSV infections in Porto Alegre occurs from June to September.^2,14^ Despite knowing the seasonal behavior of BVA in some Brazilian capitals, we lack a mapping covering the entire national territory, with the purpose of indicating AVB seasonal peak periods in each state.

RSV transmission takes place through eye or nasal contact with contaminated secretions, and the virus can be recovered after more than one hour on infected surfaces.^3^ The incubation period can last five days, and viral infection varies according to disease intensity and the child’s immune status.^15^ In addition to preventive measures to avoid contagion, there is a drug for the prevention of AVB,^16^ Palivizumab. It is not a vaccine, but an immunoglobulin—an antibody—that induces specific passive immunization against RSV. Immunization with Palivizumab is made in monthly intramuscular doses of 15 mg/kg/dose during epidemic months.^3^ Usually, this medication is administered in five doses during the five months of highest incidence of the virus, as it lasts for 30 days.^5^ The effectiveness of passive immunization is 1/200, reducing hospitalization rates for AVB by 55%.^3^

Due to the high costs, Palivizumab is restricted to greater risk cases.^5,17^ Therefore, it is essential to carry out studies that identify the period of the year in which the highest incidence of AVB cases occurs in each Brazilian state and/or region, with the purpose of indicating the best month to start medication according to local characteristics, enabling an efficient funding in the fight against AVB.

That being said, this study aims to identify the AVB seasonal period by analyzing a time series of hospitalization rates between 2000 and 2019 in each Brazilian state and the Federal District, in order to suggest the best month to start immunization with Palivizumab. Classic time series models were adjusted and used to predict monthly rates of hospitalizations for AVB in 2020.

## METHOD

This time series consists of children younger than 12 months, born between January 2000 and December 2019 and diagnosed with AVB, distributed across 26 Brazilian states and the Federal District. The number of hospitalizations for AVB and the number of live births between 2000 and 2019 were obtained from the website of the Department of Informatics of the Unified Health System in Brazil (DATASUS). Then, the monthly rates of hospitalizations for AVB per 10,000 children were calculated based on the ratio between monthly hospitalizations for AVB and annual live births. Rates were multiplied by 10,000 to facilitate interpretation of results. Data on AVB hospitalization were accessed with the International Classification of Diseases, 10th revision — ICD J21.0, J21.8 and J21.9, which comprise AVB caused by RSV, other specified microorganisms, and unspecified cases.

To assess the significance of the monotonous linear trend of hospitalizations for AVB from 2000 to 2019 in different Brazilian regions, the Cox-Stuart trend test was applied. In addition, the incidence (in %) of hospitalizations for AVB between 2000 and 2019 was calculated for each Brazilian region based on the ratio between total hospitalizations for AVB and total live births in the region. The result was multiplied by 100 to obtain the percentage incidence. Thus, considering the incidences (in %), two-proportion comparison tests were used to verify the possible influence of children’s biological sex on the rates of hospitalizations for AVB in each region. Furthermore, the test of comparison of multiple proportions by the method of Benjamini and Yekutieli^24^ was conducted to compare incidences of hospitalizations for AVB in different Brazilian regions.

Then, the time series modeling process based on the Autoregressive and Moving Average (ARMA) class models and their extensions was carried out on the series of hospitalization rates by AVB in the 26 Brazilian states and the Federal District, to predict the behavior of the series for the months of 2020.

The ARMA class models, proposed by Box and Jenkins (1970), are capable of modeling stationary time series, that is, series that do not show behavioral patterns over time and develop randomly around a constant average.^19^ ARMA models are a cautious alternative to describe time series involving a minimum number of parameters.

In general, real time series present in non-stationarity form, such as a trend. In order to model a trended time series, an extension of the ARMA model class is used, the Autoregressive Integrated Moving Average (ARIMA) models.^19^ The difference of ARIMA models is the “integration” of a step in which successive differences occur in the series of interest, transforming it in a stationary series. In addition to a graphic analysis of the time series, it allows to identify the behavior of an increasing or decreasing linear trend through hypothesis tests such as the Cox-Stuart test (signal test).^18^

Another form of recurrent non-stationarity in real series is seasonality. To model time series with seasonality, another extension of the ARMA model class is used: the Seasonal Autoregressive Integrated Moving Average (SARIMA) models.^19^ In addition to simple successive differences intended to remove a trend, these also create seasonal differences in order to remove seasonality and obtain stationarity. The process of modeling time series with models of the ARMA class and its extensions occurs firstly by identifying a SARIMA model for each location, following the usual procedure by Box and Jenkins (1970).

The number of simple differentiations d and seasonal differentiations D (s=12) is selected to obtain a stationary series. Once the series become stationary, the sample autocorrelation (SAC) and partial sample autocorrelation (PSAC) functions are used to specify the order p of the polynomial of autoregressive terms and the order q of the polynomial of terms of moving-averages, necessary for the adequate representation of the time series being modeled.^17,18^ The next step is to estimate the parameters by the maximum likelihood method, since the ***ε***_***t***_ ***ε***_***t***_ are normally distributed.^20^ The model selection was aided by information such as Akaike Information Criterion (AIC) and Bayesian Information Criterion (BIC), likelihood ratio test, lower error variance and parsimony. To verify the adequacy of the model, the Ljung-Box tests were used to detect autocorrelation and the Jarque-Bera tests to verify the normality of residuals. Finally, a prediction was made.^18,19^ This study took place as recommended by Resolution 466/2012 of the National Health Council. The project was approved by the Research Ethics Committee of Universidade Estadual de Maringá (Opinion 739.422/2014), and the Free and Informed Consent Term was not used, since data were obtained from secondary sources. The analysis was performed using R language^21^. In preparing data, the packages read.dbc, dplyr, data.table, Hmisc and tidyr were used; For the adjustment and forecasting of the series, the forecast package was used; maps were designed through XML packages, RCurl, maptools, RColorBrewer, maptools and Maps.

## RESULTS

The study cohort comprised 615,657 children under 12 months of age diagnosed with AVB. Of these, 367,580 (60%) were males and 248,077 (40%) were females. In addition, 449,395 (73%) children were younger than six months and, 98,442 (16%) being boys and 67,820 (11%) being girls. On the other hand, 166,262 (27%) children were older than six months, of which 269,138 (44%) were boys and 180,257 (29%) were girls. The children diagnosed with AVB were distributed according to the five regions of Brazil. Of the total, 51,524 (8%) lived in the North Region, 107,237 (18%) in the Northeast Region, 288,715 (47%) in the Southeast Region, 125,331 (20%) in the South Region and 41,954 (7%) in the Midwest Region.

After categorizing the interval between 2000 and 2019 into four periods (2000 to 2004, 2005 to 2009, 2010 to 2014 and 2015 to 2019), we could see the increase in hospitalizations for AVB over the periods, both in terms of percentage and of estimated annual trends, which were statistically significant (p<0.0001) in all Brazilian regions, as shown in Table 1. In the South Region, there was an increase of approximately one case every three years per 10,000 live births.

**Table 1.** Number of cases and proportion by period, age group and sex compared to the total number of cases per Brazilian region. Total cases, annual trend and incidence by region from 2000 to 2019.

Timeframe	Sex	Age	North		Northeast		Midwest		Southeast		South	
			n	%	n	%	n	%	n	%	n	%
2000-2004	F	<6	2,002	3.89	4,913	4.58	2,614	6.23	16,083	5.57	7,114	5.68
		≥6	1,125	2.18	2,000	1.87	1,070	2.55	4,073	1.41	2,491	1.99
	M	<6	2,948	5.72	7,767	7.24	4,096	9.76	24,473	8.48	10,590	8.45
		≥6	1,593	3.09	2,806	2.62	1,549	3.69	5,819	2.02	3,336	2.66
2005-2009	F	<6	3,066	5.95	7,201	6.72	2,697	6.43	19,798	6.86	8,186	6.53
		≥6	1,800	3.49	2,594	2.42	988	2.35	5,606	1.94	2,865	2.29
	M	<6	4,875	9.46	10,726	10.00	3,812	9.09	29,355	10.17	12,187	9.72
		≥6	2,608	5.06	3,765	3.51	1,482	3.53	8,239	2.85	4,137	3.30
2010-2014	F	<6	3,751	7.28	8,369	7.80	2,879	6.86	23,696	8.21	9,693	7.73
		≥6	2,655	5.15	3,239	3.02	1,476	3.52	7,779	2.69	4,190	3.34
	M	<6	5,694	11.05	12,971	12.10	4,388	10.46	36,012	12.47	14,652	11.69
		≥6	3,956	7.68	4,776	4.45	2,021	4.82	11,492	3.98	5,947	4.75
2015-2019	F	<6	3,782	7.34	10,052	9.37	3,480	8.29	29,304	10.15	11,066	8.83
		≥6	2,608	5.06	4,101	3.82	1,706	4.07	10,465	3.62	4,824	3.85
	M	<6	5,484	10.64	15,654	14.60	5,114	12.19	41,502	14.37	16,838	13.43
		≥6	3,577	6.94	6,303	5.88	2,582	6.15	15,019	5.20	7,215	5.76
2000-2019 total			51,524		107,237		41,954		288,715		125,331	
Annual trend			0.24*		0.22*		0.06*		0.30*		0.37*	
Incidence			0.83^†^		0.62^†^		0.91^†^		1.24^†^		1.60^†^	

*Statistical significance obtained by Cox-Stuart test and estimated linear trend from 2000 to 2019; †Incidence (%)from 2000 to 2019, obtained by ((100*Total_Cases_Region))/(Live_Births_Region), statistically different by the test of comparisons of multiple proportions using the method of Benjamini and Yekutieli.^24^

Using the two-proportion comparison test to examine the incidence of AVB hospitalizations according to sex, a statistically significant difference was found for all regions (p<0.0001), with a higher incidence among males. According to Table 1, this occurs mainly in children younger than six months of age and, over the periods, this percentage shown an upward trend. Regarding the incidence of hospitalizations due to AVB between 2000 and 2019 in different Brazilian regions, statistical significance was also found by the multiple proportion comparison test with the Benjamini and Yekutieli^24^ method (p<0.0001), noting that the South region has the highest incidence, followed by the Southeast, Midwest, North and Northeast regions.

In identifying the model to be adjusted, given the general presence of seasonal effect, seasonal differentiation was adopted for the 27 series of monthly rates of hospitalizations by AVB, transforming them into stationary series and providing access to their autocorrelation in the SAC and PSAC. After identifying a model for each series, the parameters were estimated, the suitability of the model was verified and the behavior of the time series was predicted for the months of 2020.

**Figure 1.**
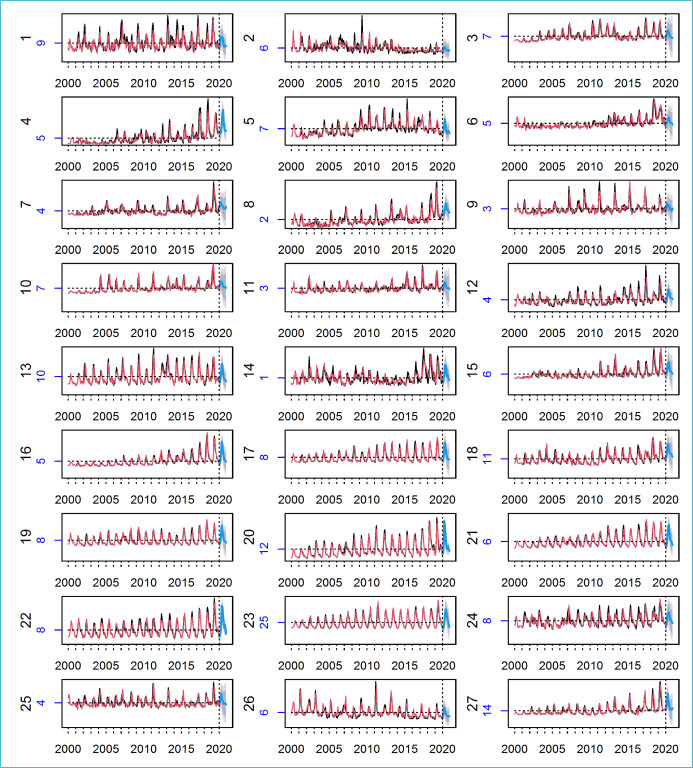
Original series (black), fitted models (red) and forecast (blue) with 12 steps ahead for each Brazilian state and the Federal District. The numbers on the left side of each graph correspond to the identification of States (black) and the average monthly rates of hospitalizations for AVB between January 2000 and December 2019 (blue).

**Chart 1.**
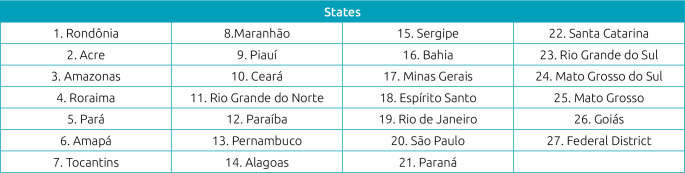
Identification of the 26 Brazilian states and the Federal District.

The adjustment and forecast of SARIMA models in the series of the 26 Brazilian States and the Federal District are shown in Figure 1. The vertical dashed line separates the adjustment (red) and forecast (blue) periods, composed of 12 steps forward. In addition, the numbering on the left side of each graph corresponds to the identification of States from Chart 1.

The number that follows the horizontal dotted line, in blue and also located on the left side of each graph, refers to the average monthly rates of hospitalizations for AVB between January 2000 and December 2019.

The monthly rates of hospitalizations for AVB per 10,000 live births in Brazil for 2020 are shown in Table 2. In order to highlight the months with the highest rates and facilitate the identification of the best time of year for immunization with Palivizumab, the shaded region in Table 2 indicates the five highest expected hospitalization rates for each state and the Federal District. In general, the period between March and July 2020 had the highest predicted rates of hospitalizations for AVB, indicating the temporal location of the seasonal peak.

**Table 2.** Forecast of monthly rates of hospitalizations for acute viral bronchiolitis for 2020 in the 26 Brazilian states and the Federal District. The five highest predicted rates for each state and the Federal District are highlighted in gray.

State	Jan	Feb	Mar	Apr	May	Jun	Jul	Aug	Sep	Oct	Nov	Dec
RO	5.47	6.05	13.66	12.4	13.08	13.62	10.71	6.96	5.31	6.99	6.20	6.22
AC	2.18	2.87	7.44	6.19	6.57	6.43	4.85	3.95	3.43	4.20	3.26	3.84
AM	5.29	6.55	10.32	12.40	13.51	11.17	8.48	6.89	5.72	6.27	5.76	5.30
RR	9.77	9.83	10.25	11.31	18.48	30.21	33.91	21.82	15.53	12.58	12.36	12.92
PA	4.53	4.73	7.76	9.82	13.21	9.71	5.49	4.30	5.56	5.97	5.78	4.59
AP	4.35	4.73	6.19	7.75	9.69	11.37	9.25	6.00	5.38	5.11	5.05	3.56
TO	6.35	7.9	10.23	10.35	7.93	7.97	6.55	5.67	5.50	5.63	5.56	6.81
MA	4.09	3.67	3.54	3.94	5.14	6.53	6.71	5.17	4.74	5.04	4.21	4.11
PI	2.16	2.55	5.64	5.23	5.14	4.04	4.68	2.58	3.11	2.51	2.04	3.17
CE	8.20	10.04	13.39	14.38	14.94	12.28	9.7	7.68	8.18	8.56	7.76	7.27
RN	2.88	2.84	4.33	5.95	7.17	5.81	4.36	3.49	3.27	3.27	3.48	2.85
PB	2.90	3.27	5.36	6.63	9.67	9.09	7.13	5.81	5.17	5.02	4.89	4.57
PE	6.94	5.24	7.92	13.14	20.85	19.87	13.02	9.21	7.83	8.12	7.08	6.24
AL	0.90	0.49	1.64	1.89	3.65	3.74	3.04	1.69	1.89	1.81	1.00	1.10
SE	4.75	5.41	8.77	15.54	19.22	18.27	12.87	9.16	8.95	8.02	6.92	5.98
BA	3.70	2.74	5.80	11.91	20.6	17.57	11.23	8.42	6.1	6.14	4.62	4.51
MG	4.79	4.21	11.31	21.43	26.06	20.71	14.02	8.52	6.72	6.59	5.96	5.85
ES	11.2	12.87	23.52	33.48	33.33	24.43	20.81	19.33	19.36	18.86	15.95	13.98
RJ	3.92	3.90	6.64	16.66	26.04	20.64	14.61	8.76	7.86	7.15	6.88	5.49
SP	8.68	7.54	20.77	41.61	35.33	25.43	16.51	12.03	12.26	15.25	11.95	9.75
PR	2.09	2.20	5.33	12.75	19.62	20.68	14.11	8.48	7.13	7.10	5.22	3.58
SC	2.29	2.87	5.60	13.10	26.00	25.18	18.78	11.35	8.74	11.47	7.92	4.54
RS	6.07	4.67	10.77	18.99	46.64	68.82	66.5	45.57	35.46	30.03	17.87	9.93
MS	4.84	4.31	8.72	13.05	13.98	14.09	11.61	8.51	8.62	7.88	6.51	5.69
MT	2.96	3.33	7.12	10.08	7.00	5.63	4.41	3.19	3.50	3.56	3.25	2.74
GO	2.25	5.04	10.23	9.75	7.71	6.21	4.87	3.88	3.41	3.66	3.45	3.67
FD	17.87	19.34	46.83	61.59	41.3	29.44	22.77	17.07	16.61	17.39	17.06	20.21

**Figure 2.**
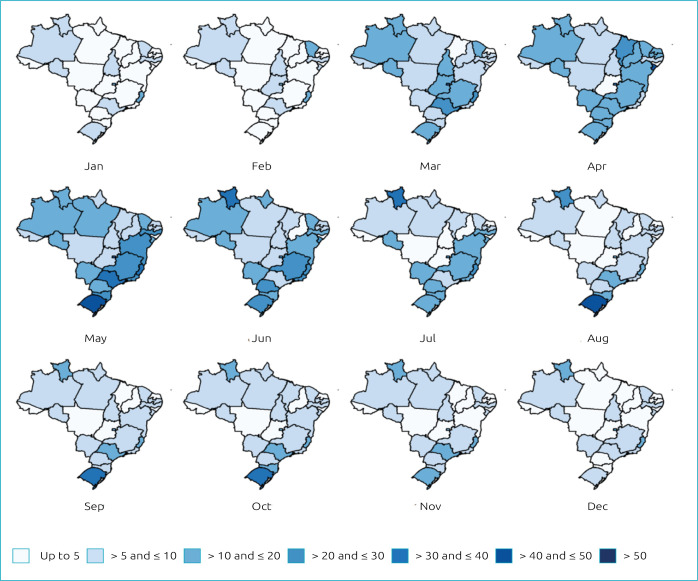
Map of monthly rates of hospitalizations for acute viral bronchiolitis predicted for 2020 in each Brazilian state.

The expected AVB hospitalization rates for 2020 were categorized into seven hierarchical intervals: up to 5, >5 and ≤10, >10 and ≤20, >20 and ≤30, >30 and ≤40, >40 and ≤ 50 and finally >50. The intervals are represented in Figure 2 by blue scales that darken as the rates of hospitalizations for AVB go up. Figure 2 is a spatial representation of the rates of hospitalizations by AVB predicted for 2020, from Table 2. The spatial representation helps one view and identify Brazilian states to be more or less affected by the AVB in 2020.

The darkest scales are concentrated between March and July 2020, like in Table 2, temporally locating the peak of hospitalization rates for AVB in Brazil in 2020. In addition, the South Region had the highest rates of hospitalizations for AVB predicted for 2020, while the States of the Northeast Region had the lowest rates.

In the South Region, the State of Rio Grande do Sul stood out with the highest rates of hospitalizations for AVB predicted for 2020. Its seasonal peak had the longest duration among all States, with high rates from May to October 2020, during fall, winter and part of the spring. Its rates range from 35.46 to 68.82 hospitalizations for AVB per 10,000 live births. In the Northeast Region, the State of Alagoas had the lowest rates predicted for 2020, reaching a maximum of 3.74 hospitalizations per 10,000 live births. Furthermore, the states of Amazonas and Pernambuco showed predicted rates of hospitalizations for AVB lower than 22 hospitalizations per 10,000 live births.

## DISCUSSION

This study verified the increase in incidence of hospitalizations for AVB over 2000 through 2019 in all Brazilian regions. The South Region had the highest rates of hospitalizations for AVB predicted for 2020. The seasonal peak of hospitalizations expected for 2020 in the State of Rio Grande do Sul comprised the fall, winter and part of the spring, with rates ranging from 35.46 to 68.82 hospitalizations per 10,000 live births.

In all Brazilian regions, male children aged less than six months had a higher incidence of hospitalizations for AVB. Studies show that boys develop respiratory tract infections more often than girls, with the exception of sinusitis, otitis externa, and probably tonsillitis. Anatomy and behavioral/socioeconomic characteristics may explain this difference between genders. There is evidence that the peripheral airways are disproportionately narrower during the first few years of life in boys, which may predispose them to lower respiratory tract infections.

It is worth mentioning that this research is naturally subject to some limitations, since it works with secondary data. One of them is the non-immediate updating and possible incompleteness of the data available on the DATASUS platform.^22^ There is also a lack of coverage of all hospitalizations in the country, as the data stored in the Hospital Information System refer to hospitalizations funded by the Unified Health System in public or private hospitals. Another limitation stems from the possible underreporting of hospital admissions, which depend on each federative unit.^23^

In France, hospital records from 2009 were analyzed in search of cases of hospitalizations for AVB of children under one year of age and factors associated with death. Cases were described based on the variables: age, sex, underlying conditions (including bronchopulmonary dysplasia, cystic fibrosis, and congenital heart disease), length of hospital stay, recurrent hospitalizations, intensive care unit (ICU) admission, and use of assisted ventilation. The hospitalization rate was 35.8 per 1,000 children under one year of age in 2009. Approximately 10% of hospitalized children required treatment in ICU.^24^

In Brazil, an analysis of the seasonality of hospitalization rates for bronchiolitis and pneumonia resulting from RSV between 2005 and 2012 suggests the beginning of Palivizumab prophylaxis in January for the North, Midwest and Southeast regions, in February for the Northeast region, and in March for the South Region.^5^

Studies addressing the prevalence and circulation of RSV in children with acute respiratory diseases in different states point to a greater circulation of the virus from April to May in the Southeast, North, Northeast and Midwest regions.^5,24,25^ In the South Region, the peak of RSV infections occurs later, between June and July,^5^ concomitant to the influenza season. In this study, coincidentally, the forecast for the peak of hospitalization rates for AVB in Brazil in 2020 was between March and July, starting with fall, in March, and covering part of the winter in the months of June and July.

It is important to consider that the public data available on DATASUS is delayed by up to two years. Thus, the use of 2020 data is not suitable because they are partial and still being entered into the system. Even if they were available, it would be necessary to develop an interrupted series analysis or intervention analysis, since an external event (pandemic) influenced the occurrences and records of the outcome. After two years of the pandemic, with daycare centers and schools open again and the relaxation of social isolation, the previous pattern of RSV tends to establish itself again over time. Anticipating occurrences is of great importance, especially for public management. The present study is not limited to a forecast for 2020, but indicates the historical pattern of regions and periods/months of the year at greatest risk. If a period/month was identified, it means the pattern has become evident over the years. The most critical months are different in each region—another reason for attention and more accurate decision-making, enabling optimization and cost reduction with the administration of the monoclonal antibody and with hospitalizations.

An epidemiological surveillance system to monitor RSV activity is of utmost importance for the implementation and maintenance of passive immunization programs with monoclonal antibodies in risk groups. Since there is a well-defined pattern of viral circulation, knowing local epidemiological data, considering regional climatic characteristics and paying attention to eventual changes over the years and the emergence of new pathogens, leads to better management of RSV prevention programs.

## References

[1] Monteiro AI, Bellei NC, Sousa AR, Santos AM, Weckx LY (2014). Respiratory infections in children up to two years of age on prophylaxis with palivizumab. Rev Paul Pediatr..

[2] Freitas AR (2014). Impact of influenza and respiratory syncytial virus on mortality and hospitalizations and Implications for Public Policies in Brazil [tese].

[3] Carvalho WB, Johnston C, Fonseca MC (2007). Acute bronchiolitis, an updated review. Rev Assoc Med Bras (1992).

[4] Albernaz EP, Menezes AM, César JA, Victora CG, Barros FC, Halpern R (2003). Risk factors associated with hospitalization for bronchiolitis in the post-neonatal period. Rev Saude Publica..

[5] Freitas AR, Donalisio MR (2016). Respiratory syncytial virus seasonality in Brazil: implications for the immunisation policy for at-risk populations. Mem Inst Oswaldo Cruz..

[6] Sociedade Brasileira de Pediatria (2011). Diretrizes para o manejo da infecção causada pelo Virus Sincicial Respiratório (VSR).

[7] Farhat CK, Cintra OA, Tregnaghi MW (2002). Vaccination and the respiratory tract--what should we know?. J Pediatr (Rio J)..

[8] Leader S, Kimmie K (2003). Recent trends in severe respiratory syncytial virus (RSV) among US infants, 1997 to 2000. J Pediatr..

[9] Calegari T, Queiroz DA, Yokosawa J, Silveira HL, Costa LF, Oliveira TF (2005). Clinical-epidemiological evaluation of respiratory syncytial virus infection in children attended in a public hospital in midwestern Brazil. Braz J Infect Dis..

[10] Nascimento JP, Siqueira MM, Sutmoller F, Krawczuk MM, Farias V, Ferreira V (1991). Longitudinal study of acute respiratory diseases in Rio de Janeiro: occurrence of respiratory viruses during four consecutive years. Rev Inst Med Trop Sao Paulo..

[11] Vieira SE, Stewien KE, Queiroz DA, Durigon EL, Török TJ, Anderson LJ (2001). Clinical patterns and seasonal trends in respiratory syncytial virus hospitalizations in São Paulo. Brazil. Rev Inst Med Trop S Paulo..

[12] Freitas AR, Francisco PM, Donalisio MR (2013). Mortality associated with influenza in tropics, state of São Paulo, Brazil, from 2002 to 2011: the pre-pandemic, pandemic, and post-pandemic periods. Influenza Res Treat..

[13] Moura FE, Borges LC, Souza LS, Ribeiro DH, Siqueira MM, Ramos EA (2003). Hospital study of acute respiratory infections in children of Northeast Brazil. J Bras Patol Med Lab..

[14] Straliotto SM, Siqueira MM, Machado V, Maia TM (2004). Respiratory viruses in the pediatric intensive care unit: prevalence and clinical aspects. Mem Inst Oswaldo Cruz..

[15] Collier L, Oxford J, Kellam P (2016). Human virology.

[16] Toma TS, Venancio SI, Martins PN, Sato HK (2013). Uso profilático de palivizumabe na prevenção de infecção pelo vírus sincicial respiratório em crianças de alto risco. Boletim do Instituto de Saúde..

[17] Franco J, Costa C, Queiroz AM, Dias Alves A, Virella D, Jorge A (2006). Estimativa da eficiência do uso de Palivizumab na prevenção de hospitalizações por infecção por vírus sincicial respiratório numa coorte de prematuros. Repositórios Científicos de Acesso ABerto de Portugal..

[18] Morettin PA, Toloi C (2006). Análise de séries temporais.

[19] Vandaele W (1983). Applied time series and Box-Jenkins models.

[20] Gottman JM (1981). Time-series analysis: a comprehensive introduction for social scientists.

[21] R Core Team (2020). R: A language and environment for statistical computing.

[22] Machado JP, Martins ML, Leite IC (2016). Quality of hospital databases in Brazil: some elements. Rev Bras Epidemiol..

[23] Correia LO, Padilha BM, Vasconcelos SM (2014). Methods for assessing the completeness of data in health information systems in Brazil: a systematic review. Cienc Saude Colet..

[24] Benjamini Y, Yekutieli D (2001). The control of the false discovery rate in multiple testing under dependency. Ann Statist..

[25] Bezerra PG, Britto MC, Correia JB, Duarte MC, Fonceca AM, Rose K (2011). Viral and atypical bacterial detection in acute respiratory infection in children under five years. PLoS One..

